# Prevalence of Obesity and the Factors Associated with Low Obesity Awareness among Urban Adolescents in Harare, Zimbabwe

**DOI:** 10.3390/nu15102302

**Published:** 2023-05-13

**Authors:** Ashleigh Pencil, Tonderayi M. Matsungo, Nobuko Hongu, Naomi Hayami

**Affiliations:** 1 Graduate School of Human Life Science, Osaka City University, 3 Chome-3-138 Sugimoto, Sumiyoshiku, Osaka 558-8585, Japan; 2 Department of Nutrition, Dietetics and Food Sciences (DNDFS), University of Zimbabwe, Mt Pleasant, Harare P.O. Box MP167, Zimbabwe; tmatsungo@gmail.com; 3 Graduate School of Human Life and Ecology, Osaka Metropolitan University, 3 Chome-3-138 Sugimoto, Sumiyoshiku, Osaka 558-8585, Japan; kay.hongu@gmail.com (N.H.); hayami@omu.ac.jp (N.H.)

**Keywords:** adolescents, overweight, obesity, awareness, perceptions, Zimbabwe

## Abstract

Obesity is a global health problem. In developing countries such as Zimbabwe, obesity is both an emerging health problem and a grey area, particularly among adolescents. This study assessed the prevalence of obesity and factors associated with low obesity awareness among adolescents. Method: A cross-sectional survey was performed using an interviewer-administered questionnaire. The participants were 423 adolescents aged 14–19 years recruited from 10 schools in Harare using a stratified random sampling technique. Data were analyzed using SPSS software (version 23) and binary logistics regression was used to examine the factors associated with low obesity awareness. The level of significance was set at *p* < 0.05. Results: The median± IQR age was 16 (14-18) years, and overweight and obesity affected 15.8% of the participants with higher proportions among girls (73.1%, *p* = 0.002). Low obesity awareness was observed in 27.1% of the adolescents with a higher proportion among girls (67.0%, *p* = 0.001), 14–16-year-olds (51.3%, *p* = 0.317), and obese adolescents (56.7%, *p* = 0.001). Significant factors associated with low obesity awareness were household heads lacking formal education [OR = 9.41 (2.20–40.36), *p* = 0.003] and inadequate (poor) food habits [OR = 2.58 (1.33–5.01), *p* = 0.005]. Conclusions: Our study showed that adolescents had different obesity awareness levels and diverse perceptions in terms of obesity causes, and a range of potential solutions. Obesity awareness and nutrition education should address adolescents’ poor eating habits while taking cognizance of the different education levels of household heads.

## 1. Introduction

The rise in obesity poses a challenge to public health worldwide. Overweight and obesity are significant public health concerns, with approximately 5% to 16.5% of adolescents in Africa being obese [1]. Zimbabwe is one of the countries with a rising and unresolved obesity prevalence of 36.6% among adolescents since 2015 [2]. Obesity leads to an increase in non-communicable diseases (NCDs) such as type 2 diabetes mellitus (T2DM), cardiovascular diseases, and hypertension, which are currently part of Zimbabwe’s biggest health threats [3]. Zimbabwe is experiencing a nutrition transition where the consumption of obesogenic foods is high in urban areas and energy-dense foods associated with western lifestyles have been adopted [4]. The causes of obesity are multifactorial, including individual, environmental, and societal factors. Socio-cultural perceptions and beliefs fuel the increase in overweight and obesity [5]. In many African countries including Zimbabwe, it is commonly believed that healthy people should not be skinny as it symbolizes poverty and ill health [5,6,7].

The perception that being overweight is a good sign of health and prosperity is accepted in Zimbabwe and other African countries such as South Africa and Morocco [4,8]. In the African context, mothers are encouraged to eat more for their well-being and that of their infant after childbirth; this belief results in excessive weight gain [9]. Furthermore, mothers/guardians often fail to recognize unhealthy weight among children and adolescents [10]. Therefore, it is important to design obesity awareness programs for adolescents, especially girls, before they become mothers. The adolescent period is a critical time for altering physical activity, dietary patterns, and nutrition knowledge to avoid excessive weight gain [10,11]. Thus, weight management remains an important health challenge for adolescents, especially in Zimbabwe where there is a stigma attached to being “thin”, as the labeling of thin individuals is often associated with being HIV-infected. The same stigma has also been reported in South Africa and Botswana [8,12,13]. Furthermore, the adolescent age group is often left out in many nutrition programs, hence weight gained in childhood may continue to adolescence and adulthood [4].

Tackling obesity may require other strategies, such as understanding the individual’s perceptions of overweight and obesity [14]. Obesity perceptions are important in the field of health promotion and behavior change as they measure awareness levels which contribute to prevention and management strategies [15]. Assessing obesity awareness among adolescents is important because obesity increases with age; furthermore, adolescence is a stage of pre-independence where eating habits including snacking after school or when hanging out with friends, skipping meals, and making food choices are not always monitored by their parents [15,16,17]. High schools in Harare enroll students aged 13–19 [18]. These schools do not offer school lunch; therefore, it is a common practice for adolescents to buy snacks at break and lunch time, and after school. It is known that when adolescents select snacks, they select based on taste over nutrition, which could lead to overweight and obesity [19]. They more often choose salty, crunchy foods as snacks and sweet beverages over healthier alternatives such as fruit or water [16]. More importantly, adolescents are at a greater risk of emotional eating. It is believed that during adolescence, pubertal hormones begin to influence appetite and body weight [20]. Therefore, obesity awareness among adolescents could be an important intrinsic motivator towards making healthy food choices and eating habits.

This study was guided by the health belief model (HBM) to understand adolescents’ beliefs and perceptions about obesity. In the context of obesity, HBM has three categories that lead to health behavior change. Modifying factors (1) include age, gender, body mass index (BMI), food habits, nutrition knowledge, and physical activity. Individual beliefs (2) include perceived susceptibility to obesity, perceived severity, perceived barriers to obesity prevention, and perceived self-efficacy. Action cues (3) include obesity intervention program design and implementation. The HBM states that people’s beliefs influence their health-related actions or behaviors and readiness to take action; this depends on the person’s ability to understand their susceptibility, the severity of the threat, their ability to bring the desired change (self-efficacy), and barriers to change (if they exist) [21]. It is reported that obesity misperceptions are severe in children and adolescents [22,23]. Therefore, understanding their perceptions may increase our understanding of how they may respond to weight reduction interventions. Thus, this study assessed the prevalence of adolescents’ perceptions on various issues related to obesity, and the factors associated with low obesity awareness among adolescents in Harare, Zimbabwe. The knowledge of adolescents’ obesity awareness and perceptions contributes to the framework for obesity prevention strategies and intervention programs for adolescents and the general population.

## 2. Materials and Methods

### 2.1. Study Design and Theoretical Framework

The study was carried out in Harare, the capital of Zimbabwe. The city has an area of 940 km^2^ and a population of 15,178,979 based on the 2022 census [24]. The participants were adolescents aged 14 to 19 years attending secondary schools in Harare. Harare has 299 high schools with a total of 355,633 learners [18].

### 2.2. Sample Size and Sampling Technique

The sample size was calculated using the Dobson formula [25] where Z-value = 1.96, *p* is the prevalence of obesity based on a previous study [26], and c is the confidence interval = 0.95. A sample size of 480 adolescents was found to be sufficient, and after a 10% attrition adjustment, the final sample size was 432. The stratified random sampling technique was used to select ten high schools from the registry of The Ministry of Primary and Secondary Education. The schools were further divided into strata based on their geographical locations and socio-economic zones (high, intermediate, and low), class level (form 2 to form 6) based on the Zimbabwean education system, and age groups (14–16 years and 17–19 years). Sampling weights were then applied to adjust for gender and non-response.

During recruitment, recruited participants were asked to remain in the classrooms, and the researcher provided information to all prospective participants regarding the study and its objectives, what participation would entail, including the measurement of weight and height for BMI estimation, waist and hip circumference measurements, and the length and duration of the self-administered questionnaire. Prospective participants were informed that no incentive for participation would be offered, and there were no penalties for discontinuing participation. Each student could drop out of the study at any time during the administration of the questionnaire. The participants were asked to take their consent forms home for the parents/guardians to sign. After recruitment, the investigator coordinated with staff members for them to collect the signed consent forms, and the participants were given the date and time for the administration of the questionnaire within their classrooms. After the collection of signed consent forms, 423 in-school adolescents were successfully enrolled in this study. On the questionnaire administration day, a team of research assistants was always present during the administration of the questionnaire to clarify possible doubts and answer questions.

### 2.3. Data Collection and Tools

#### 2.3.1. Structured Questionnaire

An interviewer-administered questionnaire was used to collect data on the adolescents’ socio-demographics, food habits, nutrition knowledge, and physical activity levels (PAL). The questionnaires were adapted to collect data on obesity perception (OP) scores (perceived susceptibility, severity, and benefits of obesity prevention) [27], self-efficacy and barriers to change [28], nutrition knowledge scores (NKS) [29], food habits scores (FHS) [30], and physical activity scores (PAS) [31], see Appendix A. The final questionnaire had seven sections. Socio-demographic and anthropometry (10 questions), obesity perceptions (15 questions), self-efficacy (8 questions), barriers to change (9 questions), nutrition knowledge (20 questions), food habits (23 questions), and physical activity (7 questions).

#### 2.3.2. Obesity Awareness

Obesity awareness measures were evaluated and categorized based on the degree to which adolescents’ obesity perceptions were assessed. Multiple choice or Likert-scale questions of obesity perceptions were used to create total scores. These scores were used to categorize the obesity awareness variable into low (OP total scores <50%) and high (OP total scores ≥50%) groups. Obesity awareness was defined as low and high awareness using existing theoretical models and previously validated scales of illness awareness, its core domains, and psychometric properties of other health conditions [32,33,34]. An example of an obesity perceptions question is, “How many years does obesity shorten an individual’s life expectancy?”

#### 2.3.3. Self-Efficacy and Barriers to Change

This questionnaire was adapted from [27]. The self-efficacy (SE) section aimed at estimating how each student can assume attitudes and behaviors that can improve his or her health status related to nutrition. The total score was 24 and was categorized as low (SE < 50%) or high (SE ≥ 50%). The barriers to change (BtC) questions assessed the knowledge and perceptions on challenges that individuals have faced or will face in trying to modify dietary eating habits. The BtC total score was 18 and was categorized as minor (BtC < 50%) or major (BtC ≥ 50%). Examples of SE and BtC questions are, “Do you think you can lose or gain weight if needed?” and, “Do you know how to improve your diet?”, respectively.

#### 2.3.4. Nutrition Knowledge, Food Habits, and Physical Activity Levels

##### Nutrition Knowledge

This questionnaire was adapted from [29]. NKS was categorized as inadequate (NKS <50%) or adequate (NKS ≥ 50%). The instrument was a practical and easy-to-administer tool with acceptable reliability in high school students. This section had three subscales: adequate and balanced nutrition, essential nutrients, and malnutrition-related diseases. Items consists of complete sentences of correct or incorrect statements. Cronbach’s alpha coefficient was 0.85 overall. Examples of nutrition knowledge questions are, “Regularly eating breakfast improves school performance” and, “Obesity may be due to excessive fat consumption” with (true, false, and not sure) answer options.

##### Food habits

This questionnaire was adapted from [30]. The FHS was calculated as follows:

$$FHS=No.\;of\;healthy\;responses\times (\frac{23}{No.}of\;items\;completed)$$

where inadequate was (FHS < 50%) and adequate was (FHS ≥ 50%). This AFHC had an internal reliability of Cronbach’s α = 0.82. Examples of food habits questions are, “I try to ensure I eat plenty of fruit and vegetables” and “I often eat sweet snacks between meals?”

##### Physical activity

This questionnaire was adapted from [31]. Physical activity score (PAS) responses were structured in different ways according to each question, each score ranging from 1 to 4, with the maximum score assigned to the healthiest habit. The total score of the PA section was 28; this was categorized as inadequate (PAS < 50%) or adequate (PAS ≥ 50%). It had an internal reliability Cronbach’s alpha of 0.71. Examples of food habits questions are, “Do you usually practice any form of physical activity?” and “What do you prefer doing during your free time?”

#### 2.3.5. Anthropometry

Height was measured to the nearest 0.1 m using the stadiometer (Leicester^®^ Height Measure, Seca, Birmingham, UK). Weight was measured using an electronic bathroom weighing scale (Sunbeam, Cape Town, South Africa), and waist and hip circumferences using the Seca 201 measuring tape (Seca, UK). The nutritional status of the children and adolescents (5–19 years) was determined using WHO standard protocols [34]. The WHO AnthroPlus software to BMI-for-age Z-scores (BMIAZ) and Height-for-age Z-scores (HAZ). Waist circumference (WC) ≥90th percentile for children and adolescents is defined as central obesity [35]. Waist–hip ratio (WHR) was classified as abnormal in males if the ratio was ≥0.9 and ≥0.85 in females. Waist-to-height (WtHR) was classified as an indicator of a high risk of central obesity if the ratio was ≥0.5 [36].

### 2.4. Data Analysis

The data were analyzed using IBM SPSS Statistics for Windows v23 (IBM Corp., Armonk, NY, USA) and the normality of the data was checked using Shapiro–Wilk tests and Q-Q plots. The continuous variables were transformed into categorical variables where applicable. The relationship between the categorical variables was assessed using Pearson’s Chi-squared test with Bonferroni adjustments, and in cases where cell counts were below five, Fisher’s exact test was used. Factors associated with obesity awareness were explored with binary logistic regression analysis using the conditional backward elimination method. The choice of the model of best fit was determined by comparing the Nagelkerke R^2^ and the Hosmer and Lameshow test. The variables entered in Step 1 are as follows: gender, age, location, education level of the household head (HH), employment HH, BMI, self-efficacy, barriers to change, physical activity, food habits, and nutrition knowledge.

## 3. Results

### 3.1. Socio-Demographic Characteristics

The socio-demographic characteristics of the participants are summarized in Table 1. The median and IQR range for the participants was 16 (14-19) years. The majority of the participants were girls (53.2%, *p* = 0.001) and in the 14–16 years age group (54.1%, *p* = 0.317). Most of the adolescents came from average-sized families (84.9%, *p* = 0.272), and lived in high-density locations (59.8%, *p* = 0.630) with both parents (66.7%, *p* = 0.253), whose household head had tertiary education (57.2%, *p* = 0.001) and was formally employed (53.9%, *p* = 0.010).

### 3.2. Obesity Awareness and Socio-Demographic Characteristics

More girls than boys had low obesity awareness (67.0%, *p* = 0.001), and more 14–16-year-olds also had low obesity awareness (51.3%, *p* = 0.317). However, there was no significant difference between the age groups. In the BMI category, 56.8% of the overweight/obese adolescents had low obesity awareness (*p* < 0.001). The education level of HH and employment status were significantly associated with low obesity awareness. Further analysis revealed that the significance was specifically on the HH with tertiary education (35.7%, *p* < 0.001) and formally employed (55.7%, *p* = 0.010), respectively (Table 1).

#### Obesity Perceptions Overview

**Figure 1:**
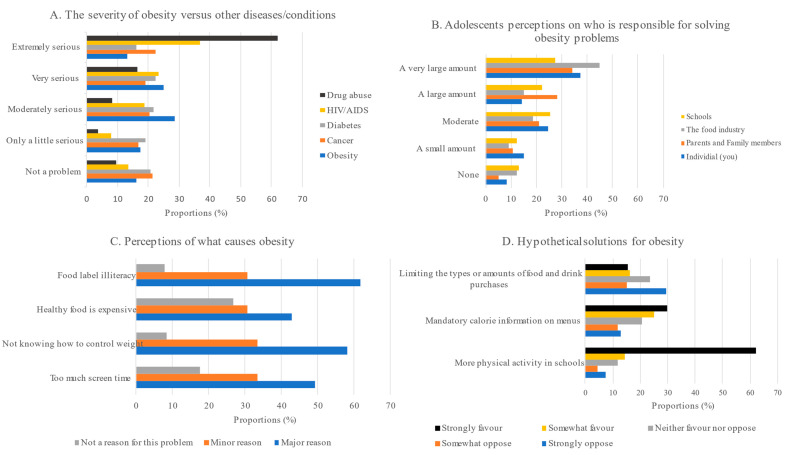
Adolescents’ perceptions of the seriousness of overweight and obesity problem in Zimbabwe.

Figure 1 shows adolescents’ perceptions of various issues related to obesity. Compared to other diseases and health-related issues such as cancer, diabetes, HIV and AIDS, and alcohol and drug abuse, only 13% of the adolescents reported that obesity was an extremely serious health issue, whereas the majority stated that obesity is a moderately serious problem (28.4%). When asked whose responsibility it was to solve the country’s obesity problem, most of them stated that the health insurance companies/medical aid companies (48.2%), and food industries (44.9%), and only 37.4% indicated that individuals are responsible. “People don’t know how to control their weight” (58.2%) and “people don’t have enough information about what’s in their food” (61.7%) were selected as the major causes of obesity (Figure 1). The adolescents strongly favored more physical activity in schools (62.2%) compared to fast-food shops showing calorie information on the menu (29.8%) and limiting the types or amounts of food and drink people can buy (15.6%) as hypothetical solutions to overweight/obesity. Lastly, when asked to describe their current weight, only 9% acknowledged that they were overweight/obese (Figure 1).

### 3.3. Obesity Awareness and Barriers to Change, Self-Efficacy, Food Habits, Nutrition Knowledge, and Physical Activity

Adolescents with more BtC had low awareness compared to those with fewer BtC (58.3%, *p* = 0.038, and those with low SE also had low awareness (56.5%, *p* = 0.005). In addition, low awareness was associated with inadequate PAS (60.9%, *p* = 0.010), inadequate FHS (61.7%, *p* = 0.017), and inadequate NKS (53.0%, *p* = 0.001). The results are summarized in Table 2.

### 3.4. Nutritional Status of Adolescents

An assessment of adolescents’ nutrition status (Table 3) revealed that obesity affected 15.8% of adolescents, with high proportions among girls compared to boys (*p* = 0.002), while the WHR (61.5%) was higher in boys than girls (*p* = 0.023), and the WtHR (central obesity indicator) was also higher among girls (72.0%, *p*= 0.005).

### 3.5. Factors Associated with Low Obesity Awareness among Zimbabwean Adolescents

The factors associated with low obesity awareness among adolescents are presented in Table 4. Significant factors associated with low awareness were HH with no formal education [OR = 9.41 (2.20–40.36), *p* = 0.003] and inadequate (poor) food habits [OR = 2.58 (1.33–5.01), *p* = 0.005].

## 4. Discussion

This study sought to assess the prevalence of overweight/obesity and factors associated with low obesity awareness among in-school adolescents in Harare, Zimbabwe. The results showed that household heads with no formal education were significantly associated with low obesity awareness among adolescents. Furthermore, inadequate (poor) food habits were also significantly associated with low obesity awareness. Interestingly, self-efficacy (SE), and barriers to change (BtC) were not significantly associated with low obesity awareness. These results were unexpected because SE and BtC are components of the HBM, which influences behavior change [32,33]. Therefore, further studies which assess adolescents’ SE and BtC in the context of obesity are required.

### 4.1. Prevalence of Overweight/Obesity and Low Obesity Awareness

Low obesity awareness, overweight/obesity, and central obesity (high WtHR) prevalence were high and more pronounced among adolescent girls. Previous studies in Zimbabwe have also reported a high obesity rate among girls compared to boys [34,35,36]. Furthermore, our results are consistent with previous studies from other African countries [37,38]. Sex differences in overweight and obesity have also been reported in other countries where biology, physical activity levels, and socio-cultural beliefs contribute to these differences [39,40,41,42]. However, to the best of our knowledge, our paper is the first one to link low obesity awareness and overweight/obesity prevalence, both of which are more pronounced in girls in Harare. These results show the vulnerability of girls compared to boys. We suggest intervention programs specifically for girls to raise obesity awareness and inform them about the importance of maintaining a healthy weight.

### 4.2. Factors Associated with Low Obesity Awareness

The lack of formal education of the household heads had a negative relationship with obesity awareness. This result is not surprising and agrees with previous studies which reported that education levels have a huge influence on diet-related diseases including obesity [43,44,45]. In an American study, an increased odds ratio in fathers’ education levels decreased the odds of their children being obese [46]. Therefore, the education levels of the parents or guardians have a bearing on the adolescents’ ability to process health information. For instance, children of more educated parents were reported to be more likely to eat breakfast, more fruits and vegetables, and fewer empty calories from snacks and sweetened beverages [46,47,48].

Contrary to this finding, a Zimbabwean study showed that having less educated parents with lower income was protective against overweight and obesity [49]. Proponents of this observation argue that in food-scarce environments, obesity should not be a problem considering that energy-dense “fast” foods are usually unaffordable and out of reach for urban, poor households (less educated parents). Interestingly, from a socioeconomic point of view, parental education and income cannot be separated [45]. The “wealth effect” is a problem in many low-income countries, including Zimbabwe, where parents tend to buy high-calorie foods packed with sugars, salt, and fats as a means of keeping up with the socio-cultural belief that fast foods/processed foods are prestigious and traditional foods represent poverty [4,6]. Therefore, our results being contradictory confirms the notion that the debate on education, wealth, and obesity remains controversial.

Nonetheless, health education targeting both high-income and low-income parents can have cascading benefits in raising obesity awareness and preventing obesity among children and adolescents [45]. This socioecological approach may prove effective considering that in most African settings, children do not have decision-making powers in terms of food purchase and preparation [7]. Therefore, educating parents to act as role models can motivate children to adopt healthy food habits and lifestyles [50]. We also speculate that obesity awareness and nutrition education in schools will help adolescents to purchase and prepare healthier foods regardless of parental influence, education, and income level.

Inadequate (poor) food habits were also significantly associated with low obesity awareness among adolescents. Food habits are conscious, collective, and repetitive behaviors, which lead people to choose, eat, and use certain foods or diets in response to economic, social, and cultural influences [51]. Poor food habits may stem from social or cultural misconceptions surrounding obesity and the failure to acknowledge its complexities [52]. In some African settings, the “earn more eat more” concept is characterized by overeating and overfeeding in support of the belief that being fat is a sign of wealth, health, and happiness [6]. An exploratory analysis revealed that 73% of the participants with low obesity awareness reported that they generally tried to have a healthy diet. However, the same individuals often skipped meals (93%) and did not eat fruits and vegetables (57.4%). These unhealthy eating behaviors are associated with overweight and obesity [53,54]. Furthermore, the dietary habits of adolescents in Zimbabwe requires further investigation within the context of socio-cultural beliefs and the changing food environments [55].

### 4.3. Cross-Cutting Issues

Binary logistic regression revealed that there were no significant associations between low obesity awareness and nutrition knowledge, physical activity levels, BtC, and BMI. These results were unexpected because of the well-known link between obesity and poor nutrition knowledge and PA [54,55]. Further research is warranted to shed more light on this area. Interestingly, our finding that a greater proportion of girls had low obesity awareness makes sense within the wider African socio-cultural context, where bigger women are considered more attractive by men [5,56,57,58,59]. Our results confirm this notion, where only 13.1% of adolescents thought that obesity is a problem in Zimbabwe.

The adolescents placed the responsibility to solve obesity mainly on food companies. Therefore, nutrition education interventions targeting this age group should prioritize messages which emphasize that the fight against obesity starts at an individual level. This is particularly important since some food companies usually prioritize profits over the health benefits of their products [60]. Therefore, people should make informed food decisions, understand how to read food labels, and ask “what’s in this food?” when they do not understand food labels [61]. There is growing evidence that nutrition decisions should be coupled with physical activity adjustments [62]. In Zimbabwe, where physical activity education is part of the school curriculum, increasing adolescents’ physical activity levels should be achieved through both organized and recreational sports activities.

## 5. Practical Implications of the Study

Zimbabwe has both established and upcoming health promotion programs [63,64,65]. Recently, the government launched the Zimbabwe School Health Policy (ZSHP), a guide for all public health, nutrition, sexual, and reproductive health related matters that affect students from preschool, to primary and high school [63]. In addition, the policy covers all aspects of the care and support provisions programs of all students, including the homegrown school feeding program. Interestingly, there are unique programs in Zimbabwean schools designed to combat chronic malnutrition (stunting), which has been exacerbated by food insecurity and deepening poverty, particularly among young children, pregnant women, and immune-compromised individuals [65]. However, despite the evidence of the double burden of malnutrition and the rise of obesity and non-communicable diseases, which are mainly caused by nutrition transition, these health promotion programs are still biased towards undernutrition with limited focus on obesity and diet-related non-communicable diseases [4,39,66]. Furthermore, there is limited nutrition programming in urban areas, in spite of the increasing urban nutrition challenges and growing population [67]. Our results contribute to the future health promotion programs and policies in Zimbabwe by establishing the base to address obesity issues among adolescents in urban areas including the capital city Harare, and we postulate that these findings may also be useful for other low-income countries.

## 6. Limitations of the Study

Several limitations should be considered when interpreting these results. Firstly, the variables in this study were developed post hoc from existing surveys. Secondly, the findings are based entirely on adolescents’ self-reports and perceptions. Therefore, we acknowledge the potential for recall bias in the estimation of food habits and any other recall-based questions. The samples were obtained from a single city in Zimbabwe, and future research should be conducted using study populations from multiple regions, in order to obtain even more accurate results than those of the present study. This study acknowledged body image concerns; however, its impact on eating behavior was not assessed. Finally, because the study was cross-sectional, the direction of causality between the variables of interest was not determined. Nevertheless, the study also had its strengths considering that adolescent nutrition and statistics is a grey area in Zimbabwe [3,4]. The current study adds to the limited literature on overweight/obesity prevalence and related factors among adolescents in low-income African countries such as Zimbabwe.

## 7. Conclusions

Our study of in-school adolescents in Harare, Zimbabwe showed obesity was prevalent and more pronounced in girls, adolescents had different obesity awareness levels, with low awareness being more pronounced in girls. They also had diverse perceptions on the complex nature of obesity in terms of causes, its seriousness, and a range of potential solutions. The findings are important for public health interventions in obesity care in Harare. Obesity awareness and nutrition education programs should address adolescents’ eating habits, especially among girls, while taking cognizance of the different education levels of household heads by using mass media programs to raise more awareness of the causes, consequences, and preventive measures, while hammering misconceptions, to combat the growing level of obesity.

## Figures and Tables

**Table 1:** Obesity awareness across participants’ socio-demographic characteristics.

Variable			Obesity Awareness		
		Total *n* (%)	Low *n* (%)	High *n* (%)	*p*-Value ^1^
Sex	Boys	198 (46.8)	38 (33.0)	160 (51.9)	0.001 *
	Girls	225 (53.2)	77 (67.0)	148 (48.1)	
Age Group	14–16 years	229 (54.1)	58 (51.3)	171 (56.8)	0.317
	17–19 years	185 (43.7)	55 (48.7)	130 (43.2)	
BMI	Underweight	41 (9.7)	26 (63.4)	15 (36.6)	0.001 *
	Normal	315 (74.5)	51 (16.2)	264 (83.8)	
	Overweight/obese	67 (15.8)	38 (56.8)	29 (43.3)	
Household Size	Average	359 (84.9)	94 (81.7)	265 (86.0)	0.272
	Above Average	64 (14.8)	21 (18.3)	43 (14.0)	
Place of Residence	Low density	72 (17.0)	21 (18.3)	51 (16.6)	0.630
	Middle density	98 (23.2)	23 (20.0)	75 (24.4)	
	High density	153 (59.8)	71 (61.7)	182 (59.1)	
Family Structure	Both parents	282 (66.7)	80 (69.6)	202 (65.6)	0.253
	Single parent	79 (18.7)	16 (13.9)	63 (20.5)	
	Relatives/guardians	52 (12.3)	14 (12.2)	38 (12.3)	
	Child headed	7 (1.7)	4 (3.5)	3 (1.0)	
	Other	3 (0.4)	1 (0.9)	2 (0.6)	
Education level of HH	No formal education	19 (4.5)	12 (10.4)	7 (2.3)	0.001 *
	Primary education	15 (3.5)	9 (7.8)	6 (1.9)	
	Ordinary education	147 (34.6)	41 (35.7)	106 (34.4)	
	Tertiary education	242 (57.2)	53 (46.1)	189 (61.4)	
Employment status of HH	Formally employed	228 (53.9)	64 (55.7)	164 (53.2)	0.010 *
	Unemployed	23 (5.4)	12 (10.4)	11 (3.6)	
	Entrepreneur	172 (40.7)	39 (33.9)	133 (43.2)	

Notes: Obesity awareness: OP score <50% is low and OP score ≥50% is high. ^1^ *p*-value is Pearson’s Chi-squared test, in cases where cell values are less than 5 the Fischer’s exact test was used. * *p*-value shows significant differences (*p* < 0.05).HH = Household Head. Household size: ≤5 is average and >5 is above average. Place of residence: density describes population size.

**Table 2:** The interplay between obesity awareness and barriers to change, self-efficacy, food habits, nutrition knowledge, and physical activity.

Variable			Obesity Awareness		
		Total *n* (%)	Low *n* (%)	High *n* (%)	*p* Value ^1^
BtC	Fewer	213 (50.4)	48 (41.7)	165 (53.6)	0.038 *
	More	210 (49.6)	67 (58.3)	143 (46.4)	
SE	Low	192 (45.3)	65 (56.5)	127 (41.2)	0.005 *
	High	231 (54.6)	50 (43.5)	181 (58.8)	
PAS	Inadequate	214 (50.6)	70 (60.9)	144 (46.8)	0.010 *
	Adequate	209 (49.4)	45 (39.1)	164 (53.2)	
FHS	Inadequate	221 (52.2)	71 (61.7)	150 (48.7)	0.017 *
	Adequate	202 (47.8)	44 (38.3)	158 (51.3)	
NKS	Inadequate	171 (40.4)	61 (53.0)	110 (35.7)	0.001 *
	Adequate	252 (59.6)	54 (47.0)	198 (64.3)	

Notes: BtC (barriers to change score): <50% = fewer and ≥50%= more. SE (Self-efficacy score): <50% = low and ≥50% = high. PAS (physical activity level): adequate ≥60 min and inadequate <60 min. FHS (Food habits score): <50% is inadequate and ≥50% is adequate. NKS (nutrition knowledge score): <50% is inadequate and ≥50% is adequate. ^1^ *p*-value is Pearson’s Chi-squared test, in cases where cell values are less than 5 the Fischer’s exact test was used. * *p*-value shows significant differences (*p* < 0.05).

**Table 3:** Nutritional status of the adolescents by gender and obesity awareness.

Variable			Gender				Obesity Awareness		
		Total *n* (%)	Male *n* (%)	Female *n* (%)	*p*-Value	Totals *n* (%)	Low *n* (%)	High *n* (%)	*p*-Value
BMI	Underweight	41 (9.7)	20 (48.8)	21 (51.2)	0.002 *	41 (9.7)	26 (63.4)	15 (36.6)	0.001 *
	Normal	315 (74.5)	160 (50.8)	155 (49.2)		315 (74.5)	51 (16.2)	264 (83.8)	
	Overweight/Obese	67 (15.8)	18 (26.9)	49 (73.1)		67 (15.8)	38 (56.7)	29 (43.3)	
WHR	Normal	349 (82.5)	166 (44.7)	205 (55.3)	0.023 *	371 (87.8)	99 (86.1)	272 (88.3)	0.535
	High	52 (12.3)	32 (61.5)	20 (38.5)		52 (12.2)	16 (13.9)	36 (11.7)	
WtHR	Normal	373 (88.2)	184 (49.3)	189 (50.7)	0.005 *	373 (88.2)	94 (81.7)	279 (90.6)	0.012 *
	High	50 (11.8)	14 (28.0)	36 (72.0)		50 (11.2)	21 (18.3)	29 (9.4)	

Notes: BMI (Body mass index) WHR—Waist–hip ratio: normal WHR< 0.9 and WHR 0 > 85, high; WHR ≥0.9 and ≥0.85 for boys and girls, respectively. WtHR—Waist-to-height ratio: normal (WtHR < 0.5), high (WtHR ≥ 0.5). *p*-value is Pearson’s Chi-squared test, in cases where cell values are less than 5 the Fischer’s exact test was used. * *p*-value shows significant differences (*p* < 0.05).

**Table 4:** Factors associated with low obesity awareness among adolescents.

Variable	B	S.E.	*p*-Value	Odds Ratio (OR)	95% C.I. for OR	
					Lower	Upper
Boys	−0.42	0.33	0.204	0.66	0.34	1.26
Age group (14–16 years)	−0.43	0.31	0.172	0.65	0.35	1.20
Location (LDS)	0.28	0.52	0.597	1.32	0.47	3.69
HH No formal education	2.24	0.74	0.003 *	9.412	2.20	40.36
Overweight and obese (BMI)	−0.26	0.55	0.614	0.76	0.26	2.21
Barriers to Change (More)	0.47	0.32	0.144	1.59	0.85	2.97
Physical Activity (Inadequate)	0.39	0.33	0.227	1.48	0.78	2.80
Food Habits (Inadequate)	0.95	0.34	0.005 *	2.58	1.33	5.01
NKS (Inadequate)	0.25	0.33	0.447	1.28	0.674	2.44
Constant	0.98	1.27	0.439	2.67		

Notes: Goodness of fit: Nagelkerke R^2^ = 0.362, Hosmer and Lameshow test *p* = 0.951. OR = Odds Ratio, LDS = Low-density suburbs, HH = Household, BMI = Body Mass Index, NKS = Nutrition knowledge score. * Factors significantly associated with low obesity awareness (*p* < 0.05).

## Data Availability

The datasets generated and/or analyzed during the current study are available from the corresponding author on reasonable request.

## Appendix A

Questionnaire

Please complete all the sections of the questionnaire. You must answer each item with only one choice. It is important that you complete it by yourself and do not leave any item without an answer. If you have any doubts, ask the Survey assistants. Your answers will remain anonymous, and the data collected will be used only for research.

**Section A: Socio-Demographic Information**

1. Name of school:…………………………………………
2. Age (years) [As at last birthday] :………………………….
3. Sex: [1] Male [2] Female [3] Prefer not to say
4. Where do you stay? E.g. Kuwadzana, Westgate, Hatfield:……………………
5. Who do you stay with?
[1] Both parents
[2] Single parent
[3] Relatives/Guardian
[4] Child headed
[5] Others specify……………………….
6. How many people live in the same household as you?……………………….
7. What is the employment status of your parents/guardians?
[1] Formally employed
[2] Unemployed
[3] Business owners or Self-employed
8. What is the educational level of your parents/guardians? [The household head]
[1] No formal Education
[2] Primary School
[3] Ordinary and Advanced level
[4] Tertiary level (College or university graduate)

**Section B: Obesity Perceptions**

Instructions

Please read each item carefully then, for each one circle the response that best applies to you.

1. How serious a problem is each of these health issues for adolescents in this country?

Please choose 1 response for each problem ranging from extremely serious [5], very serious [4], moderately serious [3], only a little serious [2], and not a problem [1].

	**Not a Problem**	**Only a Little Serious**	**Moderately Serious**	**Very Serious**	**Extremely Serious**
Cancer	1	2	3	4	5
Overweight and obesity	1	2	3	4	5
Diabetes	1	2	3	4	5
Alcohol and drug abuse	1	2	3	4	5
HIV/AIDS	1	2	3	4	5

2. More people are becoming obese these days. These might be the causes. For cause each, please show if you think it is a major reason, a minor reason, or not a reason for this problem.
  (A). People spend too much time in front of the TV, video games, and computer screens,
    [1] a major reason [2] a minor reason [3] not a reason for this problem
  (B). People do not know how to control their weight,
    [1] a major reason [2] a minor reason [3] not a reason for this problem
  (C). Healthy foods are expensive,
    [1] a major reason [2] a minor reason [3] not a reason for this problem
  (D). People don’t have enough information about what’s in their food,
    [1] a major reason [2] a minor reason [3] not a reason for this problem
  (E). There are not enough safe places for people to be physically active outdoors
    [1] a major reason [2] a minor reason [3] not a reason for this problem
3. Do you think one can be overweight and still be healthy?
    [1] Yes [2] No
4. How much discrimination do obese people face because of their weight?
    [1] A lot [2] A little [3] Some [4] Not very much [5] None
5. How many years does obesity shorten an individual’s life expectancy?
    [1] <5 years [2] 5–10 years [3] 11–15 years [4] 16–20 years [5] >21 years
6. Do you favour the following government policies: strongly favour [5], somewhat favour [4], neither favour nor oppose [3], somewhat oppose [2], strongly oppose [1]?

	**Strongly** **Oppose**	**Somewhat** **Oppose**	**Neither Favour nor Oppose**	**Somewhat** **Favour**	**Strongly** **Favour**
Requiring more physical activity in schools	1	2	3	4	5
Requiring take-away shops to post calorie information on menus	1	2	3	4	5
Limiting the types or amounts of foods and drinks people can buy	1	2	3	4	5

7. How much responsibility does each of the following groups have for solving the country’s obesity problems?

A very large amount of responsibility [5], a large amount [4], a moderate amount [3], a small amount of responsibility [2], or no responsibility at all [1]?

	**No Responsibility at All**	**A Small Amount of Responsibility**	**A Moderate Amount**	**A Large Amount**	**A Very Large Amount of Responsibility**
Individual (you)	1	2	3	4	5
Parents and other family members	1	2	3	4	5
Food industry	1	2	3	4	5
Schools	1	2	3	4	5
Medical aid companies	1	2	3	4	5
The government	1	2	3	4	5
State and local governments	1	2	3	4	5
Employers	1	2	3	4	5

8. Obesity increases the risk for illnesses like diabetes, high blood pressure, sleep apnoea (a sleeping disorder where breathing repeatedly starts and stops), heart disease, and cancer. Which of the following is the most effective way to treat morbid obesity?
  [1] Exercise [2] Diet control [3] Medication [4] Surgery [5] Other
9. In general, how would you rate your overall health?
  [1] Excellent [2] Very good [3] Good [4] Fair
10. Do you personally know anybody who you would consider to be obese?
  [1] Yes [2] No
11. Which of the following best describes your current weight?
  [1] Underweight [2] Normal/healthy weight [3] Overweight [4] Obese
12. How do you feel about your current weight?
  [1] Very happy [2] Happy [3] Neither happy nor unhappy
  [4] Unhappy [5] Very unhappy
13. Is it very easy, somewhat easy, neither easy nor hard, somewhat hard, or very hard to:
  (a) Get to fast-food restaurants,
    [1] It is very easy [2] somewhat easy [3] neither easy nor hard
    [4] somewhat hard [5] very hard
  (b) Find safe places to be physically active outdoors?
    [1] It is very easy [2] somewhat easy [3] neither easy nor hard
    [4] somewhat hard [5] very hard
14. When was your last visit with a doctor for a check-up?
  [1] <6 months ago [2] 6–12 months ago [3] 1–2 years ago [4] >2 years ago [5]Never
15. Has your doctor ever talked with you about the health risks of being or becoming overweight or obese?
  [1] Yes [2] No [3] I rarely go to the doctor

**Section C: Self-efficacy**

Please choose 1 answer only. Yes, no, or I don’t know.

1. Do you think you can choose anything by yourself?
[1] Yes [2] No [3] I don’t know
2. Do you think you can use advice aimed at improving your well-being?
[1] Yes [2] No [3] I don’t know
3. Do you think you can change your diet if needed?
[1] Yes [2] No [3] I don’t know
4. Do you think you can lose or gain weight if needed?
[1] Yes [2] No [3] I don’t know
5. Do you think you can use nutrition advice aimed at improving your dietary habits?
[1] Yes [2] No [3] I don’t know
6. Do you think you can use nutrition advice aimed at improving your health status?
[1] Yes [2] No [3] I don’t know
7. Do you think you can practice constant physical activity to improve your well-being?
[1] Yes [2] No [3] I don’t know
8. Do you think you can practice constant physical activity to improve your physical aspect?
[1] Yes [2] No [3] I don’t know

**Section D: Barriers to change**

Please choose 1 answer only. Either yes or no.

1. Do you have some influence on cooking food at home?
[1] Yes [2] No
2. Do you know which foods must be avoided to reduce dietary intake of fats and cholesterol?
[1] Yes [2] No
3. Do you know which foods must be restricted to reduce dietary intake of sugar?
[1] Yes [2] No
4. Do you know which foods must be eaten to increase your dietary intake of fiber?
[1] Yes [2] No
5. Do you know which benefits you could gain by eating a healthy diet?
[1] Yes [2] No
6. Do you know how to improve your diet?
[1] Yes [2] No
7. Do you know how much you must eat to satisfy your energy requirement?
[1] Yes [2] No
8. Do you know how important it is not to be influenced by your friends in choosing your food?
[1] Yes [2] No
9. Do you think that your family would support your efforts in improving your food habits?
[1] Yes [2] No

**Section E: Food habits checklist**

Please circle only 1 answer. Either True or False. In some cases, there is a third option.

1. If I am having lunch away from home, I often choose a low-fat option.
[1] True [2] False [3] I never have lunch away from home
2. I usually avoid eating fried foods.
[1] True [2] False
3. I usually eat a dessert if there is one available.
[1] True [2] False
4. I make sure I eat at least one serving of fruit a day.
[1] True [2] False
5. I try to keep my overall fat intake down.
[1] True [2] False
6. If I am buying milk, I often choose a low-fat brand.
[1] True [2] False [3] I never buy milk
7. I avoid eating lots of sausages and burgers.
[1] True [2] False [3] I never eat sausages or burgers
8. I often buy biscuits, donuts cream puffs, or cakes.
[1] True [2] False
9. I try to keep my overall sugar intake down.
[1] True [2] False
10. I make sure I eat at least one portion of vegetables or salad a day.
[1] True [2] False
11. If I am having a dessert at home, I try to have something low in fat.
[1] True [2] False [3] I don’t eat desserts
12. I rarely eat takeaway meals.
[1] True [2] False
13. I try to ensure I eat plenty of fruit and vegetables.
[1] True [2] False
14. I often eat sweet snacks between meals.
[1] True [2] False
15. I usually eat at least one serving of vegetables (excluding potatoes) or salad with my evening meal.
[1] True [2] False
16. When I am buying a soft drink, I usually choose a diet drink e.g., diet Coke.
[1] True [2] False [3] I never buy soft drinks
17. When I put butter or margarine on bread, I usually spread it thinly.
[1] True [2] False [3] I never have butter or margarine on bread
18. If I have a packed lunch, I usually include some chocolate and/or biscuits.
[1] True [2] False [3] I never have a packed lunch
19. When I have a snack between meals, I often choose fruit.
[1] True [2] False [3] I never eat snacks between meals
20. If I am having a dessert in a restaurant, I usually choose the healthiest one.
[1] True [2] False [3] I never have desserts in restaurants
21. I often have cream on desserts.
[1] True [2] False [3] I don’t eat desserts
22. I eat at least three servings of fruit most days.
[1] True [2] False
23. I generally try to have a healthy diet.
[1] True [2] False

Please recall how often you eat/skip meals.

Circle only 1 box from Never [1], Seldom [2]Sometimes [3], Often [4], Always [5]

	**Never**	**Seldom**	**Sometimes**	**Often**	**Always**
24. How often do you skip breakfast?	1	2	3	4	5
25. How often do you skip lunch?	1	2	3	4	5
26. How often do you skip supper?	1	2	3	4	5
27. How often do you eat regular meals with your immediate family at home, sitting down together?	1	2	3	4	5

**Section F: Nutrition knowledge**

*Please circle only 1 answer*

**Adequate and balanced nutrition.**
1. Regularly eating breakfast improves school performance.
[1] True [2] False [3] Not sure
2. Especially milk and eggs should be consumed at breakfast.
[1] True [2] False [3] Not sure
3. We should drink 8–10 glasses of water every day.
[1] True [2] False [3] Not sure
4. We should drink at least 2 glasses of milk every day.
[1] True [2] False [3] Not sure
5. We should consume 5 portions of fruits and vegetables every day.
[1] True [2] False [3] Not sure
6. Consuming bread and cereals (maize) is important for adequate and balanced nutrition.
[1] True [2] False [3] Not sure
7. We should not consume meat more than 3 days a week.
[1] True [2] False [3] Not sure
8. According to the nutrition expert, the amount of salt a person consumes in a day should not exceed 6 g.
[1] True [2] False [3] Not sure
9. Fast food (burgers, fried chips, fizzy drinks, etc.) is not suitable for adequate and balanced nutrition.
[1] True [2] False [3] Not sure
**Essential nutrients**
10. Nutrients are divided into six groups.
[1] True [2] False [3] Not sure
11. The carbohydrate group has more energy than the fats group.
[1] True [2] False [3] Not sure
12. Fizzy drinks contain high amounts of sugar.
[1] True [2] False [3] Not sure
13. Pasta and rice are starchy foods.
[1] True [2] False [3] Not sure
14. Chicken and eggs contain a high amount of protein.
[1] True [2] False [3] Not sure
15. Sugar beans and baked beans contain a high amount of protein.
[1] True [2] False [3] Not sure
16. Nuts are an alternative to meat in terms of protein content.
[1] True [2] False [3] Not sure
17. The most reasonable act for limiting the amount of fat is consuming biscuits.
[1] True [2] False [3] Not sure
18. Bread contains a high amount of fat.
[1] True [2] False [3] Not sure
19. Meat and chicken are important sources of omega-3 fatty acids.
[1] True [2] False [3] Not sure
20. When we consume animal fat, the amount of cholesterol in the body increases.
[1] True [2] False [3] Not sure
21. Fried chips are junk food.
[1] True [2] False [3] Not sure
22. Whole-grain bread contains more vitamins and minerals than white bread.
[1] True [2] False [3] Not sure
23. Vitamins A and C can be classified as antioxidant vitamins.
[1] True [2] False [3] Not sure
24. Green pepper and oranges contain high amounts of vitamin C.
[1] True [2] False [3] Not sure
25. Cheese contains a high amount of calcium.
[1] True [2] False [3] Not sure
26. Calcium and vitamin D are important for strong bones.
[1] True [2] False [3] Not sure
27. Meat contains a high amount of salt.
[1] True [2] False [3] Not sure
28. White bread contains more fiber than whole-grain bread.
[1] True [2] False [3] Not sure
29. Peaches do not contain a high amount of fibre.
[1] True [2] False [3] Not sure
**Malnutrition related diseases**
30. Obese people have health problems more than normal.
[1] True [2] False [3] Not sure
31. Eating fish is a risk factor for cardiovascular diseases.
[1] True [2] False [3] Not sure
32. Obesity may be due to excessive fat consumption.
[1] True [2] False [3] Not sure
33. Consuming foods such as fruits and vegetables which have high amount of fibre reduce the risk of getting cancer.
[1] True [2] False [3] Not sure
34. Reducing salt consumption does not reduce the risk of heart disease.
[1] True [2] False [3] Not sure
35. Overusing of sugar and salt is associated with health problems such as diabetes, hypertension, and heart disease.
[1] True [2] False [3] Not sure
36. The low consumption of fruits increases the risk of infectious diseases.
[1] True [2] False [3] Not sure
37. Adequate and balanced nutrition decreases the risk of anemia.
[1] True [2] False [3] Not sure

**Section G: Physical activity. (This is the concluding section)**

Please circle only 1 answer for each question.

1. Do you usually practice any form of physical activity?
[1] Always during the entire year
[2] Only in some seasons
[3] Sometimes
[4] Never
2. How many hours do you practice?
[1] 1–2 h per week
[2] 3–4 h per week
[3] More than 4 h per week
[4] Never
3. In the last 7 days, during your physical education (PE) class, how often were you very active (playing hard, jumping, and throwing)?
[1] Always [2] Quite often [3] Sometimes [4] Hardly [5] I don’t do PE
4. The physical activity you practice at school:
[1] Makes you feel well.
[2] Stimulates you to practice sports even out of school
[3] Is tiring
[4] Is boring
5. What do you prefer doing during your free time?
[1] Practicing a sport
[2] Walking
[3] Shopping
[4] Watching TV/listening to music/using the computer/reading a book
6. How many hours a day do you spend on the computer or watching TV?
[1] 1–2 h a day
[2] 3–4 h a day
[3] 5–6 h a day
[4] More than 6 h a day
7. Your lifestyle is:
[1] Very active
[2] Moderately active
[3] Sedentary
[4] Very sedentary
